# Excision of the Inferior Maxillary Bone for Osteo-Sarcoma

**Published:** 1847-01

**Authors:** William H. Deaderick

**Affiliations:** Athens, Tennessee


Art. III.—*Excision of the Inferior Maxillary bone for Osteo-Sarcoma.* By William H. Deaderick, M. D., of Athens, Tennessee.

The operation of which I propose to give a very brief account, was performed nearly thirty-seven years ago, and at that period, so far as I am informed, was unknown in surgery. Since that time it has been repeatedly executed, and the claim of having originated it has been set up by a foreign surgeon. By comparison of dates it will be seen that my operation preceded that of M. Dupuytren by two years.

On the 6th of February, 1810, Jesse Lay, a lad of about fourteen years of age, was brought to me on account of an excrescence which gradually arose from his gums, and which, in consequence of long neglect, completely enveloped the lower maxillary bone of the left side. It filled the inside of his mouth to such an extent as greatly to interfere with respiration and deglutition. Externally, the tumor exhibited the appearance of a wen of considerable size, and as it was daily augmenting it was evident that nothing short of its entire removal, with the portion of the bone it occupied, could save the life of the patient. Accordingly an incision was commenced just below the left ear, and continued along the course of the bone to the centre of the chin; a second one was made at right angles to the first. The integuments were then dissected from the tumor, and the bone sawed off at the angle of the jaw, and half an inch from the centre of the chin nearest the angle divided. The integuments were united in the usual manner, and the boy had a speedy and perfect recovery. The youth, at the time of the operation, although fourteen years of age, was not larger than boys usually are at ten or eleven; but immediately afterwards he commenced growing, and attained the ordinary stature of manhood. A well trained whisker hides, in a great measure, the scar left by the incision, and at a short distance the effects of the operation would not be observed.

Athens, Nov. 1st, 1846.

*Note.* Dupuytren is the generally accredited author of the operation above described. This distinguished surgeon removed a portion of the lower jaw for a cancerous affection of the gums in 1812. The operation of Dr. Deaderick, it will be seen, was performed two years prior to that time. Dupuytren's case was reported to the Faculty of Medicine at Paris, by Lisfranc, in 1813. The report of Lisfranc is republished in the *Dictionnaire des Sciences Medicales*, vol. xxix. p. 430. Dr. Deaderick did not give to the public any account of his operation before 1823, when he described it in the American Medical Recorder.

Dr. Mott, in a letter to Mr. Liston, has preferred a claim to priority in this operation. He says, "I claim for myself and my country *originality* in the operation of exsection of the lower jaw at the temporo-maxillary articulation, and in different proportions for osteo-sarcoma. I avow and declare solemnly that before my first exsection of the lower jaw for osteo-sarcoma, I never saw, read or heard of anything of the kind ever having been done in any country." He adds, "We repeat and aver, that the exsection of the lower jaw of even a fourth part, much less a half or two-thirds of it, for any form of sarcoma involving the whole texture of the bone, has never in our opinion been performed by any surgeon, past or present, until by myself at the time above stated."

The operation of Dupuytren is admitted not to have been for osteo-sarcoma, but for a cancerous sore situated over the angle of the jaw. Ribes, in the *Dict. des Sci. Med.*, referring to this operation, has the following words: "These facts lead to the hope that fungus, or osteo-sarcoma of the lower jaw, a disease so formidable that it has in many cases been vainly attacked with the iron and fire, will henceforward, since the operation of M. Dupuytren be removed by amputation of a portion more or less considerable of the lower jaw, without the danger of any accident, and, if the disease be local, with a certainty of success."

Many years before these predictions were uttered in Paris, the operation had been successfully performed by a young surgeon in the backwoods of Tennessee.

In a lecture delivered by Dr. Houston, of Dublin, in 1844, and published the same year in the London Lancet, the honor of having originated this operation is claimed for Mr. Cusack, who has performed it twelve times. The lecturer says, "The grand exploit of amputating the lower jaw, even from its articulations, the boldness of which has been only equalled by its success, has now become a standard operation in surgery. Persons afflicted with the distressing and loathsome disease for which this operation is undertaken, were formerly allowed to die, without any idea being entertained of the possibility of saving them; but now that a great mind, relying on a sound knowledge of the capabilities of the human frame, has set the example of extirpating the diseased mass *in toto*, many surgeons have fearlessly followed in the path thus laid open for them, and have derived honor from the success which crowned the enterprise. The success of this operation, both as regards immunity from danger, rapidity of convalescence, and the useful quality of masticatory apparatus which follows, is almost incredible."

Upon this passage Dr. Townsend, in his edition of Velpeau's Surgery, comments thus: "To whomsoever, therefore, the honor of this great triumph belongs, *mutatis mutandis*, the eulogium ought to apply equally well in Dr. Huston's conceptions, who, doubtless, would not desire to diminish one iota of it, because a name of different orthography from that of the justly respected Mr. Cusack, should happen to be found by a species of anaplastic substitution, to dovetail more completely than his with the historic facts in the case. We say cheerfully with all our heart, *palmam qui meruit ferat!*"

Dr. Deaderick's is the name which seems "to dovetail" *most* "completely with the historic facts," and to him, therefore, must the palm be awarded. True, he operated but once, and his operation was not made known to the world for many years afterwards; but it was undertaken for what appears to have been osteo-scarcoma; it involved the excision of nearly one-half of the lower jaw bone, and was crowned with perfect success. Dr. Deaderick did not call the disease *osteo-sarcoma*, but, in his account of his operation published in the Medical Recorder, described it as "a cartilaginous tumor." In the brief notice of it given above he applied no name to the affection, and the title prefixed to his communication is ours. Every medical reader knows how vague is the term "osteo-sarcoma," and what a diversity of morbid growths are called by that name. From the description of the tumor in Dr. Deaderick's case we have no doubt it would be styled osteo-sarcomatous.

It appears, then, that Dr. Deaderick preceded Dupuytren in the operation of excising the lower jaw bone two years, and that he anticipated Dr. Mott by eleven years, although he neglected to publish an account of the operation until after Dr. M. had communicated the results of his to the world; consequently Dr. M. was unapprised of what had been done by his countryman. He may still claim "for his *country*," if he cannot for himself, "originality in the operation," for Cusack's operations were performed two or three years subsequently to Dr. Mott's first. The operation has been performed by Dr. M. seventeen times. In a note appended to his letter to Mr. Liston Dr. Deaderick's operation is referred to, and this brief, obscure notice is all the allusion to it that we have found in looking through the American edition of Velpeau's great work on surgery. We have deemed it but an act of justice to a modest and worthy member of the profession to give these dates in connexion with the history of his case.

